# Patient and observer reported outcome measures to evaluate health-related quality of life in inherited metabolic diseases: a scoping review

**DOI:** 10.1186/s13023-018-0953-9

**Published:** 2018-11-28

**Authors:** Carlota Pascoal, Sandra Brasil, Rita Francisco, Dorinda Marques-da-Silva, Agnes Rafalko, Jaak Jaeken, Paula A. Videira, Luísa Barros, Vanessa dos Reis Ferreira

**Affiliations:** 1 Portuguese Association for Congenital Disorders of Glycosylation (CDG), Departamento Ciências da Vida, Faculdade de Ciências e Tecnologia, Universidade NOVA de Lisboa, 2829-516 Caparica, Portugal; 2 CDG & Allies - Professionals and Patient Associations International Network (PPAIN), Departamento Ciências da Vida, Faculdade de Ciências e Tecnologia, Universidade NOVA de Lisboa, 2829-516 Caparica, Portugal; 3 Research Unit on Applied Molecular Biosciences (UCIBIO), Departamento Ciências da Vida, Faculdade de Ciências e Tecnologia, Universidade NOVA de Lisboa, 2829-516 Lisbon, Portugal; 4 Glycomine, Inc, 953 Indiana St, San Francisco, CA 94107 USA; 5 Center for Metabolic Diseases, UZ and KU Leuven, Leuven, Belgium; 6 Faculdade de Psicologia, Universidade de Lisboa, 1649-013 Lisbon, Portugal

**Keywords:** Patient reported outcome measures (PROMs), Observer reported outcome measures (ObsROMs), Quality of life (QoL), Health-related quality of life (HrQoL), Inherited metabolic disease(s) (IMD(s))

## Abstract

**Background:**

Health-related Quality of Life (HrQoL) is a multidimensional measure, which has gained clinical and social relevance. Implementation of a patient-centred approach to both clinical research and care settings, has increased the recognition of patient and/or observer reported outcome measures (PROMs or ObsROMs) as informative and reliable tools for HrQoL assessment. Inherited Metabolic Diseases (IMDs) are a group of heterogeneous conditions with phenotypes ranging from mild to severe and mostly lacking effective therapies. Consequently, HrQoL evaluation is particularly relevant.

**Objectives:**

We aimed to: (1) identify patient and/or caregiver-reported HrQoL instruments used among IMDs; (2) identify the main results of the application of each HrQoL tool and (3) evaluate the main limitations of HrQoL instruments and study design/methodology in IMDs.

**Methods:**

A scoping review was conducted using methods outlined by Arksey and O’Malley. Additionally, we critically analysed each article to identify the HrQoL study drawbacks.

**Results:**

Of the 1954 studies identified, 131 addressed HrQoL of IMDs patients using PROMs and/or ObsROMs, both in observational or interventional studies. In total, we identified 32 HrQoL instruments destined to self- or proxy-completion; only 2% were disease-specific. Multiple tools (both generic and disease-specific) proved to be responsive to changes in HrQoL; the SF-36 and PedsQL questionnaires were the most frequently used in the adult and pediatric populations, respectively. Furthermore, proxy data often demonstrated to be a reliable approach complementing self-reported HrQoL scores. Nevertheless, numerous limitations were identified especially due to the rarity of these conditions.

**Conclusions:**

HrQoL is still not frequently assessed in IMDs. However, our results show successful examples of the use of patient-reported HrQoL instruments in this field. The importance of HrQoL measurement for clinical research and therapy development, incites to further research in HrQoL PROMs’ and ObsROMs’ creation and validation in IMDs.

**Electronic supplementary material:**

The online version of this article (10.1186/s13023-018-0953-9) contains supplementary material, which is available to authorized users.

## Background

Rare diseases are usually characterized by striking heterogeneity and complexity associated with a lack of evidence-based data and access to clinical experts. This makes it difficult for patients and caregivers to guide their decisions about disease management [1]. These factors have a tremendous impact on patients’ health-related quality of life (HrQoL) [2]. HrQoL is defined as a multidimensional concept referring to the subjective evaluation of the impact of the health status in domains related to physical, mental, emotional, and social functioning. It goes beyond direct measures of population health, life expectancy, and causes of death [3]. Patient-centred care is a recent approach in research and clinical practice [4]. One of the ways in which this concept has been advanced is through the development of patient or observer reported outcome measures (PROMs or ObsROMs, respectively). PROMs are direct reports from patients regarding their health condition registered via validated questionnaires with robust psychometric properties [5]. ObsROMs are reports made by a proxy who is in direct contact with the patient when it is not possible to obtain self-reports [6].

PROMs have numerous applications in diverse settings, including research, policy decision-making or treatment effectiveness assessment [7–9]. Besides facilitating communication between patients and clinicians (which improves healthcare provision), they can also improve patient outcomes [10]. Importantly, the use of PROMs is highly supported by regulatory agencies, such as the European Medicine Agency (EMA) and the Food and Drug Administration (FDA) [5, 11]. They are frequently used in clinical trials, mainly as surrogate endpoints [12, 13]. Among other parameters and variables, PROMs can be used to evaluate HrQoL [13–15]. While PROMs and ObsROMs are often used in common chronic conditions [16, 17], these tools have not often been applied or even developed in rare diseases, due to their specificities [6, 18, 19]. However, there are a few examples of the successful use of PROMs in rare diseases, particularly in academia, by PROM methodologists, and in collaboration with patient organizations [6, 20].

There is a need for PROMs in rare inherited metabolic disorders (IMDs), a group of more than 1000 heterogeneous and often life-threatening diseases [21]. The introduction of newborn screening programs detecting currently more than 50 IMDs and the development of new therapies, increased survival, prevalence, and improved patient outcomes [22, 23]. However, living with an IMD affects the patients’ HrQoL due to the wide range of diverse and debilitating symptoms and their chronic restrictive diets [24]. Moreover, the natural history of many IMDs is not well defined. This hampers the establishment of care guidelines. Most importantly, a curative treatment is rarely available. Subsequently, patients are generally confronted with a low HrQoL. Without adequate PROMs it is difficult to robustly monitor both symptoms and therapies in IMDs.

PROMs that specifically assess HrQoL are a particularly relevant outcome in chronic and debilitating conditions, especially because biomarkers are not always associated with meaningful benefits to the patients [25]. Until 2013, fewer than 30% of clinical studies for orphan drugs included QoL-related outcomes [26]. We reviewed the literature with three aims: (1) to identify patient and/or caregiver-reported HrQoL instruments – both generic and disease-specific - used among IMDs (2) to identify the main results of the application of each HrQoL tool and (3) to evaluate the main limitations of HrQoL instruments and study design/methodology in IMDs to guide future studies.

## Methods

### Data source and search strategy

Our scoping review strategy followed the methodological framework outlined by Arksey and O’Malley [27]. We searched the PubMed database with pre-defined search terms from inception until 26th February 2018. No other sources were included because we are a non-profit organization without external funding and, consequently, no access to databases that require subscription. Two groups of search terms (Additional file 1) were employed: 1) QoL related and 2) IMDs related terms. Free text terms were generated through a pilot PubMed search. We applied every combination of search terms from both groups connected by the Boolean operator “AND”. Resulting articles from our search were exported to Mendeley Desktop and duplicated articles were eliminated. References of relevant articles were screened, and additional articles were included by author referral.

### Study selection and data extraction

The review was conducted by two researchers. Inclusion criteria were as follows: studies had to be written in English and measure HrQoL using a PROM/ObsROM in an IMD context. Clinician reported outcomes, interviews and reviews were excluded, in order to focus on empirical research-generated evidence. Titles and abstracts were screened and studies that did not meet the criteria were excluded. The remaining article full-text versions were then read and included or excluded according to pre-defined criteria. Any disagreements were settled by discussion.

### Critical appraisal strategy

The methodological quality of included studies was assessed using a checklist (Additional file 2) with criteria adapted from a published HrQoL assessment study [28]. The main limitations of the included studies were identified resorting to this checklist.

## Results

**Fig. 1:**
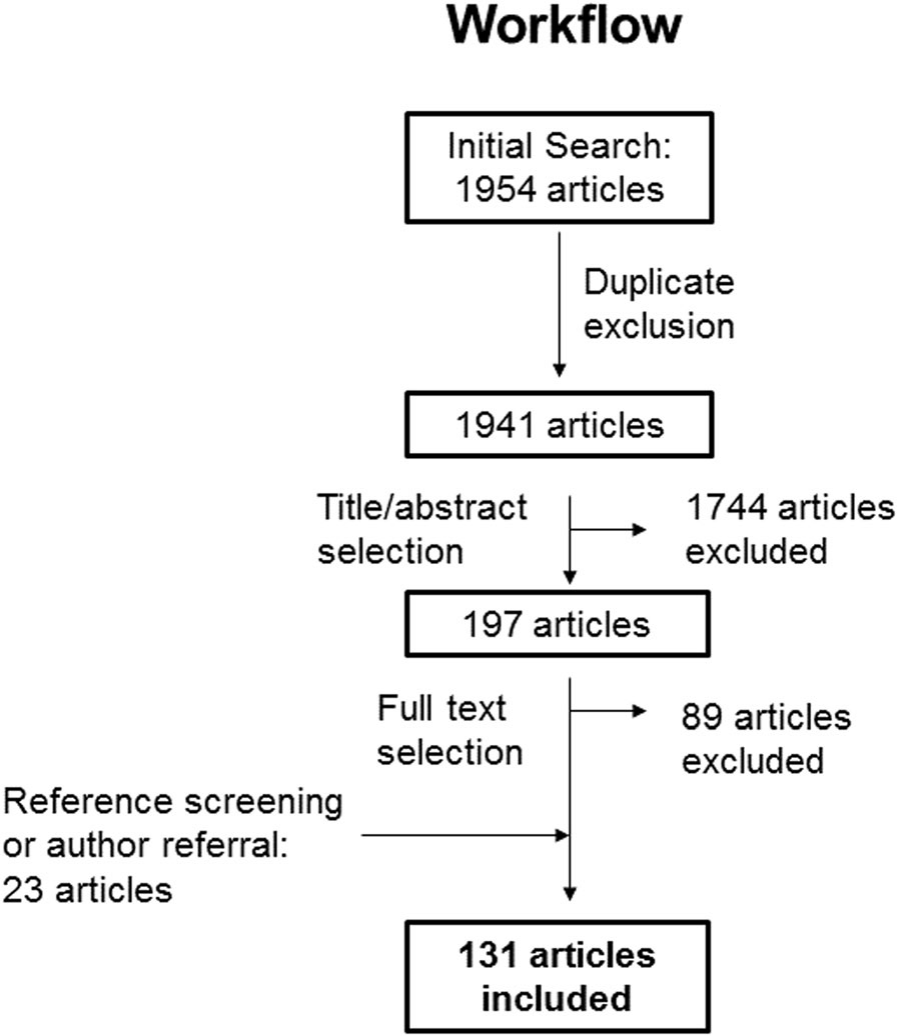
Flowchart of the literature search; Legend: Flowchart showing the workflow overview of the literature search and study selection process

The initial search of PubMed identified 1954 articles, 13 of which were duplicates. The title and abstract-based selection excluded 1744 articles. One hundred ninety-seven studies moved to the second round of selection. Based on the full-text review of each article, 91 were excluded and 108 were included. Reference screening or author referral led to the inclusion of 23 additional articles. Finally, 131 articles met the inclusion criteria. These were published between 1999 and 2017. The selection workflow is presented in Fig. 1.

### Generic and specific HrQoL instruments in IMDs pediatric and adult populations

In total, we identified 32 HrQoL assessment instruments used across IMDs (Table 1). While 84% of the manuscripts reported the use of only one HrQoL instrument, 13% applied two and another 3% used three or more instruments. The 36-Item Short Form Health Survey (SF-36) was the most frequently used measure to evaluate HrQoL across IMDs, applied in 53% of all included studies, followed by the EuroQoL- Five Dimension (EQ-5D) questionnaire (17%). Concerning the pediatric population, the Pediatric Quality of Life Inventory (PedsQL) and the Child Heath Questionnaire (CHQ) were found to be the most utilized either as self- or proxy-reports, present in 12 and 8% of the studies, respectively. Of note, the total number of instruments applied to the pediatric population is similar to that of the adult population (17 and 15 tools, respectively). However, the number of studies designed to assess HrQoL in adults is far superior to the ones directed to children. Only 2% of the studies resorted to specific instruments. Remarkably, only one of these tools is specific for adults, whereas three target children. Among the IMDs in which HrQoL was assessed, only phenylketonuria (PKU) has disease-specific questionnaires, however, there are still studies that use generic instruments to evaluate HrQoL in PKU patients. Importantly, among the identified tools there is a broader instrument targeting metabolic diseases, namely the QoL Scale for Metabolic Diseases-Parent Form. This is a validated, author-built questionnaire specifically developed to evaluate the HrQoL of children with restrictive diets [24]. Despite clear efforts, only about 7% of the more than the 1000 identified IMDs have registered patient or observer-reported HrQoL assessments.

**Table 1:** HrQoL instruments used across IMDs as PROMs or ObsROMs

Instrument Name	Sub-scales	Item number	Target Group (years)	IMDs
Adolescent- and adult-oriented instruments				
General				
15D	Mobility, vision, hearing, breathing, sleeping, eating, speech, excretion, usual activities, mental function, discomfort, depression, distress, vitality, sexual activity	15	> 16	Familial hypercholesterolemia [95]
AQOL-4D	Independent living (self-care, household tasks and mobility), relationships (friendships, isolation and family role), mental health (sleeping, worrying and pain) and senses (seeing, hearing and communication)	12	15–19 (norm)	Hereditary haemochromatosis [144]
EQ-5D	Mobility, self-care, usual activities, pain/discomfort, anxiety/depression	5 plus the VAS	> 18	Acute porphyrias [68], Fabry disease [52, 53, 88, 120–122, 127, 135, 154], hereditary hemochromatosis [155], Pompe Disease [75, 131], mucopolysaccharidosis (MPS) II [32]
EQ-5D-5 L		5 plus the VAS		MPS IVa [76, 90]
EQ-5D-3 L		5 plus the VAS		Fabry disease [74], MPS [146], MPS II [77]
EQ-5 VAS		1		Fabry disease [129, 156], Wilson disease [157]
NHP	Energy level, pain, emotional reaction, sleep, social isolation, physical abilities, work, house tasks, social functioning, sex life, interests and hobbies, vacations.	45	> 16	Pompe disease [119]
PGWBI	Anxiety, depression, positive well-being, self-control, general health, vitality	22	> 18	PKU [133]
PLC	Emotional, practical and social impact, disease-specific symptoms, diet and therapeutic impact	40	Adolescents and adults	Galactosemia [79], PKU [93]
SIP-136	Sleep and rest, eating, work, home management, recreation and pastimes, ambulation, mobility, body care and movement, social interaction, alertness behavior, emotional behavior, communication	136	Adolescents, adults and elderly	MELAS [69]
SIP-68	Somatic autonomy, mobility control, mobility range, social behaviour, emotional stability, psychological autonomy/communication	68	Adolescents, adults and elderly	Niemann-Pick disease type C [72]
SF-36	Physical functioning, role limitations due to physical health, body pain, general health, vitality, social functioning, role limitations due to emotional problems, and mental health.	36	> 14	Acute porphyrias [68], carnitine palmitoyltransferase II deficiency [158], cystinuria [143], Fabry disease [52–57, 87, 92, 107–111, 132, 141, 145, 147, 159–162], familial hypercholesterolemia [95, 112], Gaucher disease [60, 61, 98, 99, 113, 114, 128, 163, 164], GSD type I [137], hereditary hemochromatosis [67, 155, 165], mevalonate kinase deficiency [66] McArdle disease [70, 100, 101], MELAS [69], MPS [35], MPS IV [71, 115], Niemann-Pick type B [47] and type C [72], PKU [89, 94, 166], Pompe disease [62–65, 102–105, 118, 167–169], Wilson disease [116, 140, 170], X-linked hypophosphatemia [73]
SF-36-6D	Physical functioning, role limitations due to physical health, bodily pain, general health, vitality, social functioning, role limitations due to emotional problems, and mental health.	11		Pompe disease [131]
SF-12	Physical functioning, role limitations, social functioning, pain, mental health, vitality	12	> 12	Familial hypercholesterolemia [91], hypophosphatasia [80], LC-FAOD [38]
TAAQOL	Gross motor functioning, fine motor functioning, cognition, sleep, pain, social contacts, daily activities, sex, vitality, happiness, depressive mood, anger	45	> 16	Galactosemia [89], mevalonate kinase deficiency [66], PKU [46, 94]
WHOQOL-100	Vitality, psychological well-being, relationship with friends, leisure activities, relationship with parents, physical well-being, relationship with teachers, school, body image, relationship with medical staff.	100	> 18	IMDs [125], PKU [43]
WHOQOL-BREF	Physical health, psychological health, social relationships, and environment	26	> 18	Pompe disease [126], OAs, including MMA, PA, IVA and GA1; and UCDs, including CPS1 deficiency, citrullinemia type I, ASL deficiency, HHH syndrome and OTC deficiency [50], oculocutaneous albinism [81], Wilson disease [171]
Specific				
PKU-QOL Adult version	PKU symptoms, PKU in general (emotional, practical, social and overall impact), administration of Phe-free protein supplements, dietary protein restriction	65	> 18	PKU [89]
Pediatric instruments				
General				
CHQ	General health, physical functioning, role limitations due to emotional problems, role limitations due to physical health, body pain, behaviour, global behaviour, mental health, self-esteem, general health perceptions, emotional parental impact, time parental impact, family activities, family cohesion.		5–18	Familial hypomagnesemia [85], MPS IH [36], MPS II [37]
CHQ-CF87		87	5–18	Fabry disease [82], MPS II [42]
CHQ-PF28		28	5–18	PKU [89]
CHQ-PF50		50	5–18	Fabry disease [40, 82], MPS II [42], Niemann-Pick type B [47], PKU [166]
DISABKIDS-37	Independence, emotion, inclusion, exclusion, limitations, treatment	37	Children and adolescents with chronic diseases	PKU [46]
HUI 2	Sensation, mobility, emotion, cognition, self-care, pain, fertility	15 (self)	> 5	Fabry disease [40]
HUI 3	Vision, hearing, speech, ambulation, dexterity, emotion, cognition, pain	15 (self)	> 5	Fabry disease [40], MPS II [42]
KIDSCREEN-27	Walking/standing, Reach/grip, Sleeping, School/work, Activities and Breathing; and a satisfaction-with-function and a botheredness-with-function domains	27	8–18	MPS II [77]
KINDL	Physical well-being, psychological well-being, autonomy and parents, peers & social support, school environment		4–16	PKU [44, 45], carbohydrate metabolism disorders including GSD, galactosemia, and fructose-1,6-bisphosphatase deficiency; OAs including MMA, PA, MSUD, 3-methylcrotonyl CoA carboxylase deficiency and 3-hydroxy-2-methylglutaryl CoA lyase deficiency; and amino acid metabolism disorders including PKU, alkaptonuria, homocystinuria and tyrosinemia [24]
KiddyKINDL		12: self 46: proxy	4–6 or their parents	Propionic acidemia [97]
KidKINDL		24	7–13 or their parents	
KiddoKINDL		24	14–17 or their parents	
PedsQL	Physical, emotional, social and school functioning	23	5–18	PKU [43, 46, 50, 84, 89], OAs, including MMA, PA, IVA, GA1; UCDs, including CPS1 deficiency, citrullinemia type I, ASL deficiency, HHH syndrome and OTC deficiency [50], citrin deficiency [51], MPS [35], GSD type I [39], MSUD [48], nephropathic cystinosis [123], Fabry disease [83]
PedsQL parent version		23	Parents of 2–18 years old patients	Batten disease [31], citrin deficiency [51], Fabry disease [83], GSD type I [39], MMA [34], MPS [35], MSUD [48], PKU [43, 46, 84], OAs, including MMA, PA, IVA, GA1; UCDs, including CPS1 deficiency, citrullinemia type I, ASL deficiency, HHH syndrome, OTC deficiency [50] and inborn errors of metabolism in general [33]
PODCI	Upper extremity function, transfers and mobility, physical function and sports, comfort (lack of pain), happiness, satisfaction, and expectations	42	2–18	MPS IVa [115]
SF-10	Physical functioning, role-physical, bodily pain, general health perceptions, vitality, social functioning, role emotional and mental health	10	5–18 children’s parents	LC-FAOD [38]
TACQOL	General physical functioning, motor functioning, autonomy, cognition, social contacts, positive mood, negative mood	56	6–11 or their parents (parent form)	Cystinosis [86], familial hypercholesterolemia [96], galactosemia [89], PKU [30], MPS VI [49]
TAPQOL	Stomach problems, skin problems, lung problems, sleeping problems, appetite, problem behaviour, positive mood, anxiety, liveliness, social functioning*, motor functioning* and communication*	43	Parents of 9 months-6 years children	Galactosemia [89] MPS VI [49]
VSP-Ae	Gross motor functioning, fine motor functioning, cognition, sleep, pain, social contacts, daily activities, sex, vitality, happiness, depressive mood, anger	38	8–10	OAs, UCDs, MSUD [41]
VSP-A	Physical functioning, motor functioning, autonomy, cognitive functioning, social functioning, positive and negative emotional functioning	39	11–17	OAs, UCDs, MSUD [41]
VSP-Ap	Symptoms, sleeping, appetite, motor functioning, behaviour, social functioning, communication, positive and negative emotional functioning	37	Parents of patients of all ages	OAs, UCDs, MSUD [41]
Specific				
PKU-QOL	PKU symptoms, PKU in general (emotional, practical, social and overall impact), administration of Phe-free protein supplements, dietary protein restriction			PKU [89]
Child		40	9–11	
Adolescent		58	12–17	
Parent		54	PKU patients’ parents	
PKU-QOLQ	Impact, worries, satisfaction, support, well-being	NA	10–18	PKU [124]
QoL Scale for Metabolic Diseases – Parent Form	Impact of disease, attention, perception of disease, physical function, stigmatization, social support, school status, and health perception	28	Parents of children (1–15) with a metabolic disorder treated with restrictive diet for at least 1 year	Carbohydrate metabolism disorders, including GSD, galactosemia, fructose-1,6-bisphosphatase deficiency; OA including MMA, PA, MSUD, 3- methylcrotonyl CoA carboxylase deficiency, 3-hydroxy-2-methylglutaryl CoA lyase deficiency; amino acid metabolism disorders including PKU, alkaptonuria, homocystinuria, tyrosinemia [24]

HrQoL tools were divided into 2 main categories: adult-oriented and pediatric instruments. They were then further classified into general and disease-specific tools. Abbreviations: *AQOL-4D* Assessment of Quality of Life 4D, *ASL* Argininosuccinate lyase, *CHQ* Child Health Questionnaire, *CPS*1 Carbamylphosphate synthase 1, *GA1* Glutaric aciduria type I, *GSD* Glycogen storage disease, *HHH* Hyperammonemia-hyperomithinemia-homocitrullinuria, *HUI* Health Utilities Index, *MELAS* Mitochondrial encephalomyopathy, lactic acidosis, and stroke-like episode, *LC-FAOD* Long-chain fatty acid oxidation disorders, *MMA* Methylmalonic acidemia, *IVA* Isovaleric acidemia, *MPS* Mucopolysaccharidosis, *MSUD* Maple syrup urine disease, *NHP* Nottingham Health Profile, *OAs* Organic acidurias, *OTC* Ornithine transcarbamylase, *PA* Propionic acidemia, *PedsQL* Pediatric Quality of Life inventory, *PGWBI* Psychological General Well-Being Index, *PLC* Profile for the Chronically ill, *PKU-QOL* Phenylketonuria-Quality of Life, *SIP* Sickness Impact Profile, *SF*-36 36-Item Short Form survey, *TAAQOL* TNO-AZL questionnaire for adult’s HrQoL, *TACQOL* TNO-AZL questionnaire for children’s HrQoL, *TAPQOL* TNOAZL questionnaire for preschool children’s HrQoL, *UCDs* Urea cycle disorders, *VAS* Visual analogue scale, *VSP-A Vécu et Santé Percue de l’Adolescent*, *WHOQOL* World Health Organization Quality of Life

*Only for children older than 18 months

### Proxy-reports to complement self-reports

Most metabolic disorders affect not only adults but also the pediatric population. Tools adjusted to obtain information on this population, such as PedsQL and CHQ, have been developed including proxy-versions. It is a common methodological assumption that self-reports are the best method for collecting information [29], because they are the vision of oneself. In contrast, proxy-reports tend to reflect the point of view of another. However, young age and/or disease impact raise the need for proxy-reports [6]. In fact, only 6% of the analysed manuscripts registered the sole use of proxy-reports [30–37].

A combined approach of both proxy- and self-reports to obtain HrQoL data was used in 21% of the studies. For 7% of the articles analysed, there were no differences between self- and parent-proxy data [30, 38–45]. Nevertheless, parent-reported HrQoL scores were lower than reports of children with Niemann-Pick disease, MPS VI, MSUD, other OAs and UCDs. [30, 46–51[ Only in one case, in contrast, higher QoL regarding the fatigue scale was reported by parents of type II citrullinemia patients when compared with the self-reports [51].

### HrQoL is generally impaired in IMDs

A rising number of studies using self- and/or proxy reported PROMs or ObsROMs to evaluate the impact of IMDs in HrQoL has been registered. These include both observational studies and baseline measurements performed in interventional studies. The SF-36 questionnaire was the most frequently used (51% of the studies), particularly in the adult population. This instrument revealed overall HrQoL impairment in a wide range of IMDs, including Fabry disease [52–59], Gaucher disease type I [60, 61], Pompe disease [62–65], mevalonate kinase deficiency [66], hereditary hemochromatosis [67], acute porphyrias [68], MELAS [69], McArdle disease [70], MPS [35, 71], Niemann-Pick type B [47], Niemann-Pick type C [72] and X-linked hypophosphatemia [73]. The EQ-5D (15%) showed consistently low HrQoL scores in Fabry disease [52, 53, 74], Pompe disease [75], MPS [76, 77] and acute porphyrias [68]. Less frequently used tools, namely TAAQOL (6%), SF-12 (4%), PLC (4%), SIP-136 (one study) and WHOQOL-BREF (one study), detected reduced HrQoL in galactosemia (only at the mental level) [78, 79], hypophosphatasia [80], MELAS [69] and oculocutaneous albinism (only at the physical level) [81].

Regarding instruments applied to the pediatric population, the PedsQL and the CHQ were administered in 11 and 9% of the studies, respectively. They described compromised HrQoL in Fabry disease [82, 83], MPS [35, 37, 42], PKU [84], Niemann-Pick type B [47], familial hypomagnesaemia [85], MSUD [48], methylmalonic acidemia [34] and GSD type I [39]. The TACQOL (8%) and TAPQOL (4%) detected low HrQoL in cystinosis [86], galactosemia [78], MPS VI [49] and PKU (only at the emotional level) [30]. Two other tools were administered in MPS II, in one study each, namely the HUI 3 and KIDSCREEN-27, revealing a negative impact of this disease on HrQoL [42, 77].

The detrimental effect of IMDs on patients’ HrQoL is mainly attributed to disease severity and associated complications, but also to its emotional and financial burden. We found several parameters and comorbidities associated with impaired HrQoL, namely, age, kidney and respiratory function, pain, overweight, the burden of a restrictive diet as well as gastrointestinal, cardiovascular, skeletal and neurologic involvement [52, 57, 58, 60, 61, 63, 74, 76–79, 87–92].

In a set of cases, the employment of HrQoL instruments did not show substantial impairments compared to control populations. Specifically, the administration of SF-36, PLC, TAAQOL, WHOQOL-100 and KINDL (the last used in 4% of the studies) found normal HrQoL scores in PKU patients [30, 43, 45, 93, 94]. Also, in familial hypercholesterolemia patients, SF-12, TACQOL and 15D (applied in one study) did not show differences in HrQoL compared to healthy peers [91, 95, 96]. No differences in HrQoL were found in patients with mevalonate kinase deficiency when the cognition scale of TAAQOL was administered alone. Importantly, KINDL application in propionic acidemia revealed normal HrQoL scores despite poor neurological and psychosocial outcomes [97].

### HrQoL tools detect changes upon treatment in IMD

Besides clinical parameters and disease-specific biomarkers, HrQoL is now recognized as an essential instrument to determine therapeutic effects. Multiple studies included the assessment of changes in HrQoL upon treatment initiation to better evaluate the therapeutic benefits (Table 2). The SF-36 was predominantly utilized (67% of the interventional studies). Generally, this tool was capable of detecting alterations in HrQoL scores across several IMDs [60, 64, 98–114], with the exception of MPS IV and Wilson disease [115, 116]. Interestingly, whilst no changes were observed using SF-36 in late-onset Pompe disease, NHP was responsive in this patient niche [117–119]. Regarding EQ-5D, detectable variations were observed in Fabry disease patients [120–122]. However, HUI and CHQ administration in Fabry disease children did not perceive deviations in HrQoL [40]. Changes in HrQoL were captured by SF-12 in LC-FAOD. On the other hand, in this pediatric population, administration of parent-reported SF-10 did not reveal any differences [38]. In MPS IV, PODCI only detected differences in 1 out of 4 children [115]. PedsQL was able to find fluctuations in HrQoL of Fabry disease and nephropathic cystinosis patients [83, 123]. In MPS VI, the application of TAPQOL/TACQOL also detected modifications in HrQoL [49]. When HrQoL was measured using general instruments, namely TAAQOL, KINDL and PedsQL in PKU, BH_4_ responsive patients’ HrQoL did not differ from non-responsive ones 24 months after treatment initiation [44, 46]. In contrast, HrQoL assessment with the specific PKU-QOLQ instrument detected a significant improvement in the responsive patients’ life quality for up to 1 year [124].

**Table 2:** PROMs/ObsROMs HrQoL instruments used across IMDs to evaluate therapeutic benefit in clinical studies

IMD	Sample (age in years)	Treatment/Therapy	Follow up time	HrQoL tool	Effects on HrQoL and major findings	Ref.
Carnitine palmitoyl transferase II deficiency	Adults	Bezafibrate	6 months	SF-36	- ↑ HrQoL in all domains, specially role limitations due to physical health and body pain	[158]
	Children and adults (10–55)	Anaplerotic diet therapy	7–61 months	SF-36	- ↑ HrQoL in the physical component score	[106]
Fabry disease	Male adults (16–48)	Agalsidase-β	10 days-20 weeks	SF-36	- Phase 1/2 trial that proved the efficiency and safety of Fabrazyme including an ↑ HrQoL, namely regarding bodily pain, general health, vitality, physical role and emotional role domains. Placebo effects were noticed.	[107, 108]
	Adults and adolescents (> 14)	Agalsidase-β	3 years	SF-36	- ↑ HrQoL more pronounced in men	[109]
	Adults (41.4, mean)	Agalsidase-β	2 years	SF-36	- Ns changes in HrQoL	[141]
	Male adults (16–34)	Agalsidase-β	20 weeks	SF-36	- ↑ HrQoL with significant changes in the general health and the mental component score	[110]
	Adults	Agalsidade-α	2 years	EQ-5D	- Significant ↑ HrQoL	[120]
	Adults	Agalsidase-α	2 years	EQ-5D	- Significant ↑ HrQoL which negatively correlates with pain	[122]
	Adults (39.2 ± 12.3, mean)	Agalsidase-α	5 years	EuroQoL	- Significant ↑ HrQoL	[121]
	Adults (males: 44.25 ± 11; females: 52.3 ± 10.5, means)	Agalsidase-α	4 weeks	EQ-5D	- Ns differences in HrQoL in the 3 dose regimens. (NCT01218659)	[129]
	Heterozygous females (20–66)	Agalsidase-α	55 weeks	SF-36	- Phase 3 study that proved the agalsidase-α safety and efficacy in heterozygous females with significant ↑ HrQoL, mostly in the physical domain.	[111]
	Children (7–18)	Agalsidase-α	55 weeks	HUI, CHQ	- Phase 2 study that showed the efficiency and safety of agalsidase-α, however HrQoL remained unchanged. (NCT01363492)	[40]
	Adults (42.5 ± 12.5, mean)	ERT	6.1 ± 2.5 years	EQ-5D	- ↑ HrQoL in severely affected males - Unchanged HrQoL in women - Annual ↓ in HrQol in non-classical patients	[74]
	Adults (26–68)	ERT	4–7 years	SF-36	- Stable HrQoL except for the social functioning score.	[161]
	Children (6–18)	ERT	–	PedsQL	- Children on ERT had higher scores that approached significance.	[83]
	Adults (16–74)	Migalastat	2 years	SF-36	- Results did not reveal any clinical benefit (NCT00925301 and NCT01458119)	[162]
Familial hypercholesterolemia	Children and adults (9–57)	LDL apheresis	NA	SF-36	- ↑ HrQoL in 2 patients with baseline data	[112]
Gaucher disease type I	Children and adults (12–70)	Imiglucerase	4 years	SF-36	- Significant ↑ HrQoL in the physical component score and, particularly, the physical functioning, physical role limitations and bodily pain subscores. (NCT00365131)	[60]
	Adults (18–82)	Imiglucerase	2 years	SF-36	- Since baseline HrQoL approached those of the general population, there was no space for improvements and ns changes were observed.	[164]
	Adults (17–69)	Miglustat	6–24 months	SF-36	- Miglustat administration significantly ↑ HrQoL, while imiglucerase or combination of both reduced HrQoL	[113]
	Adults (35.2 ± 10.2, mean at start)	Miglustat	12–48 months	SF-36	- Similar improvements in HrQoL between miglustat and ERT-treated patients.	[114]
	Adults (18–66)	ERT	2 years	SF-36	- Self-perception of global health, physical activity and social functioning improved with ERT.	[61]
	Children and adults (> 5)	ERT or SRT	10 years (mean)	SF-36	- 65 patients achieved the therapeutic goal for HrQoL but differences between treated and untreated patients were ns.	[99]
	Children and adults (> 12) (one type 3 Gaucher disease patient)	ERT	8.5 years (mean)	SF-36	- Bodily pain was significantly decreased in ERT-naïve patients but physical functioning, role physical, general health, social functioning and role emotional scores showed clinical meaningful impairments too. - Gaucher disease patients receiving ERT have significant higher scores than Fabry disease patients also receiving ERT.	[128]
Hereditary hemochromatosis	Adults (55 ± 9.0, mean)	Erythrocytaphe-resis	2 years	SF-36	- There is no benefit in terms of HrQoL of erythrocytapheresis over phlebotomy. (NCT01398644)	[155]
MPS IH	Children and adults (2–25)	Hematopoietic cell transplant	9 years (mean)	CHQ-PF50	- Higher age at transplant correlates with poor physical scores	[36]
LC-FAOD	Children and adults (12.06 ± 13.2, mean)	Triheptanoin (UX007)	24 weeks	SF-12, SF-10	- Significant ↑ HrQoL in the physical and mental domains for adults but not for children. (NCT01886378)	[38]
MPS VI	Children and adults (5–21)	Arylsulfatase B	1.3–5-4 years	TAPQOL, TACQOL	- ↑ HrQoL regarding lung problems, sleeping, liveliness, positive mood, social functioning and communication - ↓ HRQoL in the anxiety and negative emotions domains	[49]
McArdle’s disease	Adults (18–60)	Ramipril	3 months	SF-36	-↑ HrQoL in the emotional status and social role in both ramipril and placebo groups	[100, 101]
MPS IV	Children and adults (9.8–42.2)	Elosulfase-α	48–96 weeks	SF-36, PODCI	- Stable HrQoL, except in 1 child with ↓ HrQoL (NCT01697319)	[115]
Nephropathic cystinosis	Children and adolescents (6–21)	Delayed-release cysteamine birtrate	2 years	PedsQL	- Significant ↑ HrQoL particularly in social, school and total function.	[123]
Pompe disease	Adults (41–42)	Alglucosidase-α	2 years	SF-36	- ↑ HrQoL, particularly in the bodily pain domain	[102]
	Adults (28–62)	Alglucosidase-α	52 weeks	SF-36	- 3/5 patients improved both physical and mental scores while 1/5 improved only the mental or the physical score	[103]
	Late onset adults (27–73)	Alglucosidase-α	36 months	SF-36	- Ns differences from baseline	[117]
	Late onset adults (21–69)	Alglucosidase-α	1 year	SF-36	- Ns differences from baseline	[118]
	Adults (24–76, at start)	ERT	4 years (median)	SF-36	- Significant ↑ HrQoL in the physical functioning, role physical, general health, vitality and mental health subscores, after 2 years. After 4 years, the bodily pain domain significantly worsened.	[64]
	Adult (65)	L-alanine	6 months	SF-36	- ↓ HrQoL, mainly the physical domain due to worsening of muscle function. ↑ HrQoL during placebo interval reflecting the optimism of entering a trial.	[104]
	Adults (20–71)	Exercise program	12 weeks	SF-36	- Borderline ↑ HrQoL at the mental component	[105]
	Late onset adults (35.5–60.7)	Inspiratory muscle training program	8 weeks	NHP	- Significant ↑ HrQoL exclusively in the social isolation subscore	[119]
PKU	Children and adults (4–44)	BH4	1 year	PedsQL, TAAQOL	- Unchanged HrQoL and similar between responsive and non-responsive patients	[46]
	Children and adolescents (6.6–18.7)	BH4	6 months	KINDL	- Unchanged HrQoL and similar between responsive and non-responsive patients	[44]
	Children and adults (10–49)	BH4	1 year	PKU-QOLQ	- ↑ HrQoL in responders, provisional responders and non-responders in terms of impact, satisfaction	[124]
Wilson disease	Children and adults (8–41)	Orthotopic liver transplantation	97 months (mean)	SF-36	- Ns difference between norm-based scores and patients who underwent transplantation	[116]
	Adults (36.6 ± 12.9)	D-penicillamine, trientine, zinc	8.1–12.6 years (mean)	SF-36	- D-penicillamine-treated patients had the highest HrQoL scores compared to trientine- or zinc-treated patients.	[140]

Abbreviations: *AQOL-4D* Assessment of Quality of Life 4D, *CHQ* Child Health Questionnaire, *HUI* Health Utilities Index, *LDL* Low-density lipoprotein, *NA* non available, *NHP* Nottingham Health Profile, *Ns* No significant, *PedsQL* Pediatric Quality of Life Inventory, *PGWBI* Psychological General Well-Being Index, *PLC* Profile for the Chronically Ill, *PODCI* Pediatric Outcomes Data Collection Instrument, *PKU-QOL* Phenylketonuria-Quality of Life, *SF*-36 36-Item Short Form Survey, *TAAQOL* TNO-AZL questionnaire for Adult’s HrQoL, *TACQOL* TNO-AZL Questionnaire for Children’s HrQoL, *TAPQOL* TNOAZL Questionnaire for Preschool Children’s HrQoL, *VSP-A Vécu et Santé Percue de l’Adolescent*, *WHOQOL* World Health Organization Quality of Life

**Fig. 2:**
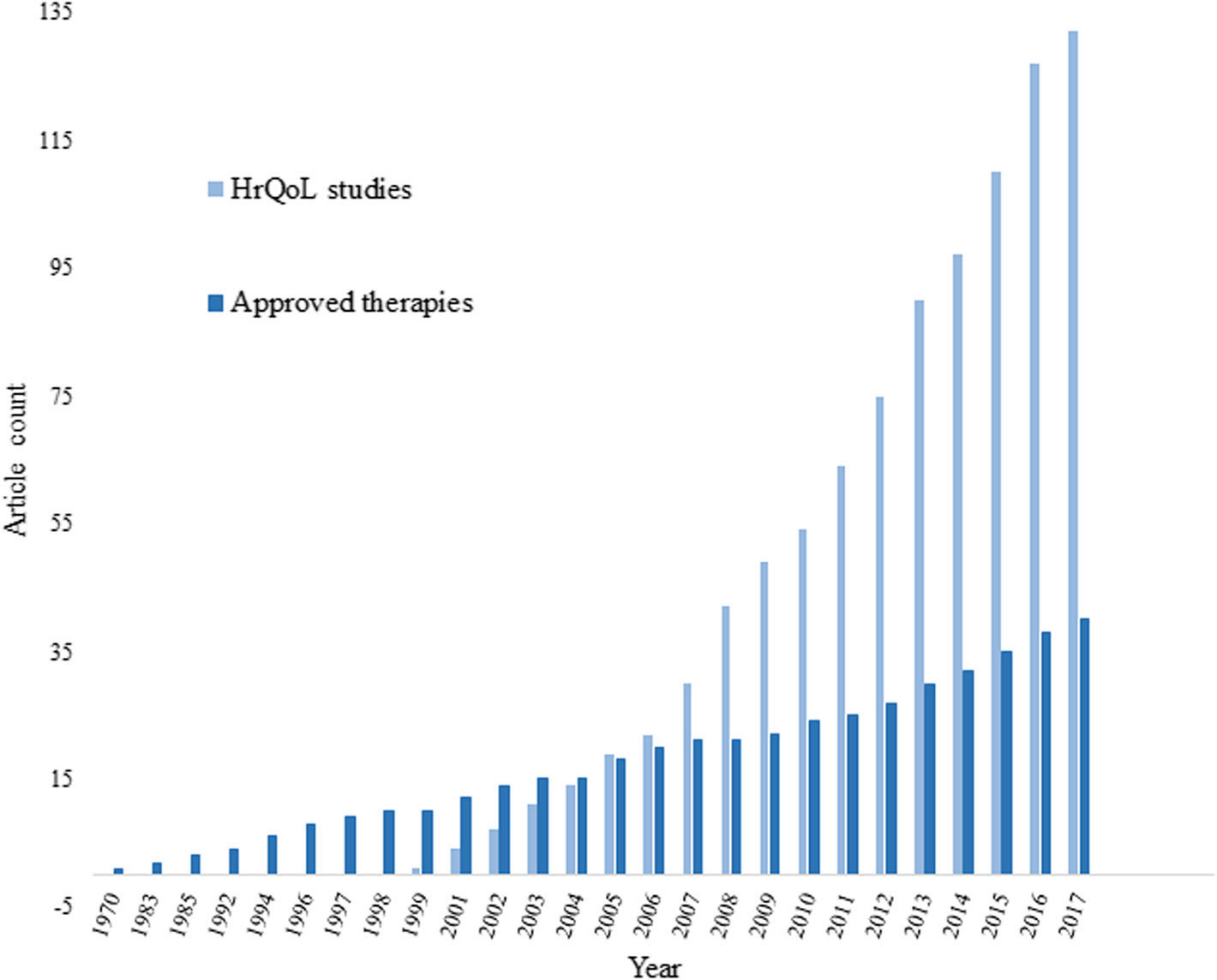
HrQoL assessment studies VS approved therapies in IMDs; Legend: Graph showing the number of HrQoL assessment studies (light blue) and approved therapies (dark blue) for the IMDs included in this review over time. The number of approved therapies is based on a specific search including EMA (http://www.ema.europa.eu), FDA (https://www.fda.gov) and Clinical Trials.gov (https://clinicaltrials.gov)

Attesting the importance of HrQoL PROMs and ObsROMs instruments, the continuous increase of HrQoL assessment reports in IMDs is accompanied by an increase in the number of approved therapies for these disorders (Fig. 2).

### Critical appraisal and main limitations of HrQoL studies

We critically assessed the quality of the included studies and identified some concerns due to an increased risk of bias (see Additional file 2). Although most studies used a standardized generic tool, validation is rarely obtained for the population in which the instrument is being used. Therefore, the results of the use of a non-specifically validated tool for a certain population should be interpreted with caution. Mostly, the problems faced by clinical researchers dealing with IMDs are inherent to the rarity of these conditions. The biggest limitation is the small sample size [34, 37, 41, 53, 56, 65, 77, 82, 85, 100, 102, 105, 112, 115, 125–130]. This makes it difficult to reach statistically significant conclusions [35, 39, 43, 44, 46, 75, 79, 83, 86, 90, 93, 99, 131–133] and also precludes the use of adequate control samples as placebo-control groups [40, 58, 70, 76, 103, 108, 109, 119, 121, 134, 135]. To counteract the problem of small samples, multinational studies are being performed. However, this can raise other limitations, particularly cross-cultural differences [42, 70, 89] as well as variations in the range of investigations, protocol and rigor between centres [92, 121]. Still, pooling data from multinational studies might be adequate if the study design, the methods, linguistic and cognitive equivalence of the concepts being measured are achieved [136]. Thus, validation should be a never-ending process and one should always look for the psychometric and cross-cultural validity, reliability and acceptability of the measure in the context of each study. Nevertheless, small sample sizes might be considered representative due to the rarity of the condition and the study objectives [39, 52, 55, 78, 89, 95]. Some countries may not have normative data available and comparison of results with foreign data or with other chronic conditions may be a source of bias [52, 54, 70, 83, 128, 131]. Moreover, 55% manuscripts do not justify the selection of the HrQoL instrument used. Since a variety of general instruments are available, this selection process should be clearly defined. Additionally, some answer option systems might not be capable of detecting small changes in health (i.e. EQ-5D) [74].

Some HrQoL studies are performed to assess the effects of a certain therapy. Consequently, they are administered before and after the therapy to provide baseline data and to register any possible improvement, respectively. In these cases, loss to follow-up due to noncompliance or abandonment is a recurrent issue [46]. Furthermore, missing data decreases the statistical validity/power of the study. Imputation of missing values can be made by researchers, but this is not always possible [137]. Finally, unreported clinical history and/or genotype data impair possible correlations with HrQoL scores [83].

Disease heterogeneity is very common to IMDs [31, 40, 75, 89, 99, 103, 115]. On the one hand, in cross-sectional studies severe forms of a disease can counterbalance the higher HrQoL scores reached by patients with less severe presentations and vice-versa [43, 61, 71, 87, 138]. On the other hand, in order to avoid erroneous conclusions drawn from differently affected patient samples, careful individual inclusion and sampling should be performed [42]. In line with this, longitudinal designs are a better approach to establish the natural history of the disease, pinpoint predictive factors of impaired HrQoL as well as to identify pre−/post-treatment alterations [41, 43, 52, 70, 139]. Nevertheless, variability in treatment or therapy duration in long-term follow-up studies [37, 41, 112, 140] as well as previous therapies and symptom management solutions can constrain long-term analysis [44, 53, 89, 105, 110, 129, 141].

Observation of changes in HrQoL over time can be difficult due to ceiling effects (i.e. patients with scores near the upper scale limit have less room for improvement) [109, 124]. In this case, high HrQoL values have been proposed to be due to (i) the disability paradox, e.g., disabled individuals reporting good HrQoL because they focus on their coping strategies and positive emotions [46, 50, 116]; (ii) patients’ expectations adaptation throughout their illness experience; (iii) adaptation by repeatedly using the same instrument [74] and (iv) variation of symptom severity [53]. Importantly, studies focusing on adult patients may often underrate the overall disease impact on HrQoL since severely affected patients may die during infancy. The spectrum of disease severity is thus not fully represented [80].

Selection bias may arise from recruitment through patient advocacy groups, health care institutions and patient registries (convenience sampling) [142]. This can cause a shift towards the inclusion of patients with either severe or milder forms of a disease who can be more or less likely to seek medical care and community support [54, 57–59, 64, 73, 76, 78, 80, 82, 92, 93, 96, 99, 143–146]. Volunteer participation and the patient-reported character of the questionnaires may indirectly exclude patients with poor literacy or cognition [79]. Further inherent drawbacks to patient-reported data include non-verification of diagnosis and symptomatology in medical records [31, 58, 63, 80, 143, 144, 146], recall bias [31, 34, 70, 147] and social desirability [70].

## Discussion

Nowadays, regulatory agencies such as FDA, EMA or health technology assessment bodies are turning to HrQoL PROMs data to support decision making. However, PROMs are not yet used routinely in clinical practice. These tools can provide natural history data, but also clinical endpoints for therapeutic trials. Consequently, there is a rise of HrQoL assessment studies using PROMs or ObsROMs in rare IMDs to fill in this gap. This reinforces the importance and need for these instruments to accelarate research and effective clinical solutions.

We identified 32 instruments used either to assess patients’ HrQoL or to evaluate the risk or benefit of a specific treatment. Most of the instruments found are generic since for most IMDs, specific instruments are still inexistent. The predominant use of SF-36 is probably due to its validity in an extensive group of populations, languages, and the fact that it comprises a wide age range (14 years and older) [148]. The second most used tool was the EQ-5D, mainly within adult populations. Recently, the EuroQoL group developed the EQ-5D-Y, the child-friendly version of EQ-5D, which might increase the use of this instrument. However, until today, no population norms using EQ-5D-Y have been published. There are considerable fewer studies focused on assessing HrQoL in children. The lack of natural history studies in this population, the perception of a better health, the inability to respond for themselves, and the fact that there are much less pediatric clinical trials might be contributing factors. Nevertheless, the PedsQL and the CHQ were used in 12 and 8% of the studies respectively. The fact that these instruments can be used either as PROMs and/or ObsROMs might contribute to their frequent use compared to other pediatric tools. Frequently, studies with a combined approach of self- and proxy-reports are carried out in this population. This is even more common in disorders presenting cognitive developmental disability. Also important is the reduced ability of young children to identify issues in emotional functioning [83]. Proxy reports can accurately reflect the same aspects as observed in patients. However, they can also reflect the parent’s state of mind, fears and doubts regarding their children, which results in lower HrQoL scores [46]. Therefore, self and proxy HrQoL data should be acquired and compared.

Due to the great symptom heterogeneity found among IMDs, we cannot however grade the 32 instruments found accordingly to their appropriateness to use in these disorders. Therefore, the research team should always look for the conceptual design of each instrument and analyse if it is suitable for the features of the patient population under assessment.

Generic instruments present some advantages. They can be applied to every disease or clinical manifestation and allow comparisons between different patient groups or between patients and healthy populations. However, they are not directed to the features of each condition. This may omit meaningful clinical outcomes limiting the study power [36, 46, 47, 50, 62, 64, 77, 147]. Disease-specific instruments include relevant questions related to a particular disease and thus are more responsive and sensitive [149]. However, they only confer the capability of making comparisons within the same patient group. Only two studies applied disease-specific tools, namely PKU-QOL and PKU-QOLQ [89, 124]. This fact further highlights the lack of specific HrQoL PROMs in the field of IMDs. As disease-specific and generic instruments assess different aspects of HrQoL, the use of both instruments in a complementary way has been suggested [150, 151]. A new group of HrQoL instruments is arising, namely disease group-specific instruments. In the metabolic field, we identified the QoL scale for Metabolic Diseases-Parent Form. More recently, a new promising but still not validated tool was developed for pediatric patients with intoxication type inherited errors of metabolism [152]. These tools focus on common aspects of different diseases, thus allowing comparisons across related but distinct patient populations. Furthermore, they are particularly important in rare diseases since they can overcome limitations associated with small sample size.

We identified instruments capable of detecting changes in HrQoL compared to normative data or following treatment/therapy initiation while others were not responsive. Additionally, in the case of Fabry adult patients, the results of the administration of SF-36 in three different studies evaluating the same therapeutic intervention diverged [109, 110, 141]. Thus, conclusions should be drawn with caution since other variables besides the quality of the intervention may influence the results. In fact, study design, sampling methods, suitability of the HrQoL instrument for a specific population and its selection according to the study characteristics are important factors to consider in order to obtain robust and reliable results. Additionally, the illness burden in several IMDs is not always easy to prove. For example, in propionic acidemia patients, no significant changes in HrQoL were found in comparison with normal individuals in spite of their poor neurocognitive and psychosocial outcomes [97]. However, we cannot exclude the fact that some IMDs affect patient’s HrQoL to a lesser degree. This has been observed in PKU [30, 43, 45, 93, 94] and familial hypercholesterolemia [91, 95, 96] after efficient treatment following early diagnosis. Nevertheless, although existing tools are not responsive in these subgroups, the measurement of the impact of a highly restrictive diet on patients’ QoL is extremely relevant and needed. In fact, PROMs are being developed on this topic to correctly evaluate the HrQoL of these patients [24].

Promising strategies to develop specific HrQoL PROMs that efficiently capture the patient’s perspective, prognosis, impactful clinical manifestations and that establish the natural history of the disease include:

- qualitative interviews with patients, their families and caregivers;
- patient registries, which also motivate patient enrolment in research projects and clinical trials [10]. The fact that Fabry disease is the condition with higher HrQoL assessment, is likely to be a direct consequence of the successful establishment of two patient registries - the Fabry Outcome Survey (NCT03289065) and the Fabry Registry (NCT00196742). Both include periodic HrQoL evaluations as a clinical outcome;
- clinical trials networks that facilitate data sharing and collaborations, ultimately improving access to the available information [153].

### Conclusion and future directions

Patient-centred approaches based on patients’ HrQoL are expanding by the implementation of PROMs or ObsROMs in clinical practice and research settings. However, this review makes it clear that they are still poorly utilized in the field of IMDs. There is a huge gap in the development of responsive disease-specific HrQoL measurement instruments that could be useful endpoints in clinical trials. To overcome the limitations inherent to the rarity of these conditions, efforts should be made not only to develop but also to adequately validate these tools. The successful establishment of international patient registry platforms might be the path with biggest potential to upgrade HrQoL studies across IMDs. They facilitate patient recruitment and uniform data collection worldwide. In line with this, the European Commission health program included a project that consists in a novel registry platform for all known IMDs – the Unified European Registry for Inherited Metabolic Disorders (https://u-imd.org/). Although there is still a long way to go as far as the proper implementation of patient-centred care is concerned, these studies and instruments are important efforts in the right direction. HrQoL assessment through PROMs and ObsROMs are an efficient way of prioritizing the patient perspective. They drive research and more rapidly create therapeutic solutions that meet the patients’ needs and expectations.

## Additional files

Additional file 1: Search methodology: List of the terms used to search the Pubmed database and search strategy. (DOCX 18 kb)

Additional file 2: Article critical appraisal: 19 items checklist for the critical appraisal of the included studies. (DOCX 71 kb)

## Abbreviations

(Hr)QoL: (Health-related) quality of life
CHQ: Child Health Questionnaire
EMA: European Medicine Agency
EQ-5(3)D: EuroQoL five(three)-dimension
FDA: Food and Drug Administration
IMD(s): Inherited metabolic disorder(s)
P(Obs)RO(M/s): Patient (observer) reported outcomes (measure/s)
PedsQL: Pediatric Quality of Life Inventory
PKU: Phenylketonuria
SF-36: 36-Item Short form health survey
