# Supplementary material for: Patient and observer reported outcome measures to evaluate health-related quality of life in inherited metabolic diseases: a scoping review
Source: Orphanet J Rare Dis. 2018 Nov 28;13:215. doi: 10.1186/s13023-018-0953-9 (PMC6263554; doi:10.1186/s13023-018-0953-9)
Supplement: Supplementary file 1 — Search methodology: List of the terms used to search the Pubmed database and search strategy. (DOCX 18 kb) [file 13023_2018_953_MOESM1_ESM.docx]

**Search methodology**

1. Pubmed search terms

| **Quality of life related terms** | **IMDs related terms** |
| --- | --- |
| Quality of life **/** QoL | Hereditary metabolic diseases |
| Health related quality of life **/** HRQoL | Orphan diseases |
| Patient reported outcomes | Rare Metabolic diseases |
| Observer reported outcomes | Inborn errors of metabolism |
| Patient centred outcomes | Inherited metabolic diseases |
| Health outcomes | Rare Inborn errors of metabolism |
| Quality of life or health related quality of life assessment |  |
| Quality of life or health related quality of life survey |  |
| Quality of life or health related quality of life questionnaire |  |
| Quality of life or health related quality of life tools |  |
| Quality of life or health related quality of life research |  |

1. Search strategy

‘quality of life’ related AND ‘IMDs related terms’.
