# Supplementary material for: Patient and observer reported outcome measures to evaluate health-related quality of life in inherited metabolic diseases: a scoping review
Source: Orphanet J Rare Dis. 2018 Nov 28;13:215. doi: 10.1186/s13023-018-0953-9 (PMC6263554; doi:10.1186/s13023-018-0953-9)
Supplement: Supplementary file 2 — Article critical appraisal: 19 items checklist for the critical appraisal of the included studies. (DOCX 71 kb) [file 13023_2018_953_MOESM2_ESM.docx]

**Article critical appraisal**

Table 1 – Critical appraisal checklist of the included studies. Adapted from Picot et al 2015.

| Criteria | Ali et al 2015 | Angelini et al 2009 | Arends et al 2017 | Aslan et al 2016 | Baehner et al 2003 | Barba-romero et al 2012 | Beck et al 2004 | Bik-multanowski et al 2009 | Bonnefont et al 2009 | Bosch et al 2004 | Bosch et al 2007 | Bosch et al 2015 | Bouwman et al 2011 | Brands et al 2013 | Brands et al 2015 | Bugescu et al 2016 | Cazzorla et al 2012 | Cazzorla et al 1014 | Ceravolo et al 2013 | Che et al 2016 | Cialone et al 2011 | Concolino et al 2017 | Cotugno et al 2011 |
| --- | --- | --- | --- | --- | --- | --- | --- | --- | --- | --- | --- | --- | --- | --- | --- | --- | --- | --- | --- | --- | --- | --- | --- |
| Objectives clearly stated | X | X | ✓ | ✓ | X | X | X | ✓ | X | ✓ | ✓ | ✓ | ✓ | X | X | ✓ | ✓ | X | X | ✓ | ✓ | ✓ | ✓ |
| HrQoL as primary outcome | X | X | ✓ | X | X | X | ✓ | ✓ | X | ✓ | ✓ | ✓ | ✓ | X | X | ✓ | ✓ | ✓ | X | ✓ | X | ✓ | ✓ |
| Instrument selection justified | ✓ | X | X | ✓ | X | ✓ | ✓ | X | X | X | ✓ | ✓ | ✓ | X | ✓ | ✓ | ✓ | ✓ | X | X | X | X | ✓ |
| Validated instrument | ✓ | ✓ | ✓ | ✓ | ✓ | ✓ | ✓ | ✓ | ✓ | ✝ | ✓ | ✓ | ✓ | ✓ | ✓ | ✓ | ✓ | ✓ | ✓ | ✓ | ✓ | ✓ | ✓ |
| Study design described | ✓ | ✓ | ✓ | ✓ | ✓ | ✓ | ✓ | ✓ | ✓ | ✓ | ✓ | ✓ | ✓ | ✓ | ✓ | ✓ | ✓ | ✓ | ✓ | ✓ | ✓ | ✓ | ✓ |
| Sampling method described | ✓ | X | ✓ | ✓ | ✓ | ✓ | NA | ✓ | X | ✓ | ✓ | ✓ | ✓ | NA | ✓ | ✓ | ✓ | ✓ | NA | NA | ✓ | ✓ | ✓ |
| Inclusion criteria described | ✓ | ✓ | ✓ | ✓ | ✓ | ✓ | ✓ | ✓ | X | ✓ | ✓ | ✓ | ✓ | ✓ | ✓ | ✓ | ✓ | ✓ | X | ✓ | X | ✓ | ✓ |
| Excludes relevant individuals | X | ✓ | X | X | X | X | X | ✓ | NA | X | X | X | X | X | X | X | X | ✓ | NA | ✓ | NA | X | ✓ |
| Demographics described | ✓ | ✓ | ✓ | ✓ | X | ✓ | ✓ | X | X | ✓ | ✓ | ✓ | X | ✓ | ✓ | ✓ | ✓ | ✓ | ✓ | ✓ | ✓ | ✓ | ✓ |
| Clinical features described | X | ✓ | ✓ | ✓ | ✓ | ✓ | ✓ | X | X | X | X | ✓ | ✓ | ✓ | ✓ | ✓ | X | X | X | ✓ | ✓ | ✓ | X |
| Sample size justified | X | X | X | X | X | X | X | X | X | X | ✓ | X | X | X | X | X | X | X | X | X | X | X | ✓ |
| Self-completion by participants | ✓ | ✓ | NR | NR | NR | NR | ✓ | NR | ✓ | ✓ | ✓ | ✓ | NR | ✓ | ✓ | ✓ | ✓ | ✓ | ✓ | ✓ | ✓ | ✓ | ✓ |
| Assessment time described | X | ✓ | ✓ | ✓ | ✓ | X | ✓ | ✓ | ✓ | ✓ | X | ✓ | X | ✓ | X | X | X | ✓ | ✓ | X | X | ✓ | ✓ |
| Response rate reported | ✓ | ✓ | ✓ | ✓ | ✓ | ✓ | ✓ | X | ✓ | ✓ | ✓ | ✓ | ✓ | ✓ | ✓ | X | ✓ | ✓ | ✓ | X | ✓ | ✓ | X |
| Loss to follow-up reported | NA | ✓ | NA | ✓ | ✓ | NA | X | ✓ | ✓ | NA | NA | NA | ✓ | ✓ | ✓ | NA | NA | NA | ✓ | NA | X | ✓ | NA |
| Missing data reported | X | X | X | X | X | X | X | X | X | ✓ | X | X | ✓ | X | ✓ | X | ✓ | X | X | X | ✓ | ✓ | X |
| Appropriate statistical analysis | ✓ | X | ✓ | ✓ | ✓ | ✓ | ✓ | X | ✓ | ✓ | ✓ | X | ✓ | X | ✓ | ✓ | ✓ | ✓ | X | ✓ | ✓ | ✓ | ✓ |
| Study findings described | ✓ | ✓ | ✓ | ✓ | ✓ | ✓ | ✓ | ✓ | ✓ | ✓ | ✓ | ✓ | ✓ | ✓ | ✓ | ✓ | ✓ | ✓ | ✓ | ✓ | ✓ | ✓ | ✓ |
| Study limitations described | ✓ | X | ✓ | ✓ | X | ✓ | X | ✓ | X | ✓ | ✓ | ✓ | ✓ | ✓ | ✓ | ✓ | ✓ | ✓ | X | ✓ | ✓ | X | X |

| Criteria | de Graaff et al 2016 | de Jongh et al 2003 | Demirdas et al 2013 | Douglas et al 2013 | Eggink et al 2014 | Elstein et al 2017 | Eminoglu et al 2013 | Eng et al 2001 [61] | Eng et al 2001 [77] | Eto et al 2005 | Fabre et al 2013 | Favejee et al 2015 | Feldmann et al 2017 | Gargiulo et al 2013 | Germain et al 2016 | Ghali et al 2012 | Giraldo et al 2005 | Giraldo et al 2006 | Giraldo et al 2009 | Giraldo et al 2016 | Goker-Alpan et al 2016 | Gold et al 2002 | Graesdal et al 2012 |
| --- | --- | --- | --- | --- | --- | --- | --- | --- | --- | --- | --- | --- | --- | --- | --- | --- | --- | --- | --- | --- | --- | --- | --- |
| Objectives clearly stated | ✓ | ✓ | ✓ | ✓ | X | X | ✓ | ✓ | X | X | ✓ | ✓ | ✓ | ✓ | ✓ | ✓ | ✓ | ✓ | X | ✓ | ✓ | ✓ | ✓ |
| HrQoL as primary outcome | ✓ | ✓ | ✓ | ✓ | ✓ | X | ✓ | X | X | X | ✓ | X | ✓ | ✓ | X | X | ✓ | X | X | X | X | ✓ | X |
| Instrument selection justified | ✓ | X | ✓ | ✓ | X | X | ✓ | X | X | X | X | X | ✓ | ✓ | X | ✓ | X | X | X | X | X | ✓ | X |
| Validated instrument | ✓ | ✓ | ✓ | X | ✓ | ✓ | ✝ | ✓ | ✓ | ✓ | ✓ | ✓ | ✓ | ✓ | ✓ | ✓ | ✓ | ✓ | ✓ | ✓ | ✓ | ✓ | ✓ |
| Study design described | ✓ | ✓ | ✓ | ✓ | ✓ | ✓ | ✓ | ✓ | ✓ | ✓ | ✓ | ✓ | ✓ | ✓ | ✓ | ✓ | ✓ | ✓ | ✓ | ✓ | ✓ | ✓ | ✓ |
| Sampling method described | ✓ | ✓ | ✓ | ✓ | ✓ | ✓ | NA | X | ✓ | ✓ | X | ✓ | ✓ | ✓ | ✓ | ✓ | ✓ | ✓ | X | ✓ | X | ✓ | NA |
| Inclusion criteria described | ✓ | ✓ | ✓ | ✓ | ✓ | ✓ | ✓ | ✓ | ✓ | ✓ | ✓ | ✓ | ✓ | X | ✓ | ✓ | ✓ | X | ✓ | ✓ | ✓ | ✓ | ✓ |
| Excludes relevant individuals | X | X | X | ✓ | X | X | X | X | X | X | X | X | X | NA | ✓ | X | X | NA | X | ✓ | X | X | ✓ |
| Demographics described | ✓ | ✓ | ✓ | ✓ | ✓ | ✓ | ✓ | ✓ | ✓ | ✓ | ✓ | ✓ | ✓ | ✓ | X | ✓ | ✓ | ✓ | ✓ | ✓ | ✓ | ✓ | ✓ |
| Clinical features described | ✓ | X | X | ✓ | ✓ | ✓ | X | X | X | X | ✓ | ✓ | X | X | ✓ | X | ✓ | X | X | ✓ | X | ✓ | ✓ |
| Sample size justified | X | X | X | X | X | X | X | X | X | X | X | X | X | X | X | X | X | X | X | X | X | X | ✓ |
| Self-completion by participants | ✓ | X | ✓ | ✓ | ✓ | NR | ✓ | ✓ | ✓ | NR | ✓ | ✓ | ✓ | NR | ✓ | NR | ✓ | NR | ✓ | NR | ✓ | ✓ | ✓ |
| Assessment time described | X | ✓ | ✓ | ✓ | X | ✓ | ✓ | ✓ | ✓ | ✓ | ✓ | ✓ | ✓ | ✓ | ✓ | ✓ | ✓ | ✓ | ✓ | ✓ | ✓ | ✓ | X |
| Response rate reported | X | ✓ | ✓ | X | X | X | ✓ | X | X | X | ✓ | X | ✓ | ✓ | X | ✓ | ✓ | X | X | X | ✓ | ✓ | ✓ |
| Loss to follow-up reported | NA | NA | ✓ | ✓ | NA | ✓ | NA | X | X | ✓ | NA | ✓ | ✓ | ✓ | ✓ | NA | ✓ | ✓ | ✓ | NA | ✓ | NA | ✓ |
| Missing data reported | X | X | X | X | ✓ | X | X | X | X | X | X | X | ✓ | X | X | X | X | X | X | X | X | X | X |
| Appropriate statistical analysis | ✓ | ✓ | ✓ | ✓ | ✓ | ✓ | ✓ | ✓ | ✓ | ✓ | ✓ | X | ✓ | ✓ | ✓ | ✓ | ✓ | X | X | ✓ | X | X | X |
| Study findings described | ✓ | ✓ | ✓ | ✓ | ✓ | ✓ | ✓ | X | ✓ | ✓ | ✓ | ✓ | ✓ | ✓ | ✓ | ✓ | ✓ | ✓ | X | ✓ | ✓ | ✓ | ✓ |
| Study limitations described | ✓ | ✓ | ✓ | X | ✓ | X | X | ✓ | X | X | ✓ | ✓ | ✓ | ✓ | X | ✓ | X | X | X | ✓ | ✓ | ✓ | ✓ |

| Criteria | Grunert et al 2013 | Guffon et al 2016 | Gungor et al 2013 | Gungor et al 2015 | Guran et al 2011 | Hagemans et al 2004 | Harmatz et al 2017 | Hendriksz et al 2014 | Hoffmann et al 2005 | Hoffmann et al 2007 | Hoffmann et al 2012 | Hopkin et al 2008 | Hughes et al 2013 | Hughes et al 2016 | Hyttinen et al 2008 | Jamiolkowski et al 2016 | Kanters et al 2011 | Kanters er al 2014 | Kantola et al 2012 | Kishnani et al 2009 | Koens et al 2016 | Komal-Kumar et al 2008 |
| --- | --- | --- | --- | --- | --- | --- | --- | --- | --- | --- | --- | --- | --- | --- | --- | --- | --- | --- | --- | --- | --- | --- |
| Objectives clearly stated | X | ✓ | ✓ | ✓ | X | ✓ | ✓ | ✓ | ✓ | ✓ | ✓ | ✓ | ✓ | ✓ | ✓ | ✓ | ✓ | ✓ | X | ✓ | ✓ | ✓ |
| HrQoL as primary outcome | X | ✓ | X | ✓ | X | ✓ | ✓ | ✓ | ✓ | ✓ | ✓ | X | X | X | ✓ | ✓ | ✓ | ✓ | ✓ | X | X | ✓ |
| Instrument selection justified | X | X | X | ✓ | ✓ | ✓ | ✓ | X | X | X | ✓ | X | X | X | ✓ | X | X | ✓ | X | X | X | X |
| Validated instrument | ✓ | ✓ | ✓ | ✓ | ✓ | ✓ | X | ✓ | ✓ | ✓ | ✓ | ✓ | ✓ | ✓ | ✓ | ✓ | ✓ | ✓ | ✓ | ✓ | ✓ | ✓ |
| Study design described | ✓ | ✓ | ✓ | ✓ | ✓ | ✓ | ✓ | ✓ | ✓ | ✓ | ✓ | ✓ | ✓ | ✓ | ✓ | ✓ | ✓ | ✓ | ✓ | ✓ | ✓ | ✓ |
| Sampling method described | NA | ✓ | ✓ | ✓ | NA | ✓ | X | ✓ | ✓ | ✓ | ✓ | ✓ | ✓ | ✓ | ✓ | ✓ | ✓ | ✓ | ✓ | ✓ | ✓ | ✓ |
| Inclusion criteria described | ✓ | ✓ | X | ✓ | X | ✓ | ✓ | ✓ | ✓ | ✓ | ✓ | ✓ | ✓ | ✓ | ✓ | ✓ | X | X | X | ✓ | ✓ | ✓ |
| Excludes relevant individuals | X | X | NA | X | NA | X | X | X | X | X | X | X | X | ✓ | X | X | NA | NA | NA | X | X | ✓ |
| Demographics described | ✓ | ✓ | ✓ | ✓ | ✓ | ✓ | X | ✓ | X | X | ✓ | ✓ | ✓ | X | ✓ | X | ✓ | X | X | ✓ | ✓ | ✓ |
| Clinical features described | ✓ | ✓ | X | ✓ | ✓ | ✓ | ✓ | ✓ | X | ✓ | X | ✓ | ✓ | ✓ | ✓ | ✓ | X | X | X | ✓ | ✓ | ✓ |
| Sample size justified | X | X | X | X | X | X | X | X | X | X | X | X | X | X | ✓ | X | ✓ | ✓ | ✓ | ✓ | X | X |
| Self-completion by participants | ✓ | ✓ | ✓ | NR | ✓ | ✓ | ✓ | ✓ | ✓ | ✓ | ✓ | NR | ✓ | ✓ | ✓ | ✓ | ✓ | ✓ | NR | ✓ | NR | ✓ |
| Assessment time described | X | X | ✓ | ✓ | X | ✓ | ✓ | ✓ | ✓ | ✓ | X | X | X | ✓ | ✓ | X | X | ✓ | ✓ | ✓ | ✓ | X |
| Response rate reported | X | ✓ | ✓ | ✓ | ✓ | ✓ | X | X | ✓ | ✓ | ✓ | X | ✓ | X | X | ✓ | ✓ | ✓ | X | ✓ | X | X |
| Loss to follow-up reported | NA | NA | NA | X | NA | ✓ | ✓ | NA | NA | X | NA | X | X | ✓ | X | NA | NA | ✓ | ✓ | X | NA | NA |
| Missing data reported | X | ✓ | X | X | X | ✓ | X | ✓ | X | X | X | ✓ | X | X | X | ✓ | X | X | X | X | X | X |
| Appropriate statistical analysis | X | X | ✓ | ✓ | X | ✓ | X | ✓ | ✓ | ✓ | ✓ | ✓ | ✓ | ✓ | ✓ | ✓ | ✓ | ✓ | ✓ | X | ✓ | ✓ |
| Study findings described | ✓ | ✓ | ✓ | ✓ | ✓ | ✓ | ✓ | ✓ | ✓ | ✓ | ✓ | ✓ | ✓ | ✓ | ✓ | ✓ | ✓ | ✓ | ✓ | ✓ | ✓ | ✓ |
| Study limitations described | ✓ | ✓ | ✓ | ✓ | ✓ | ✓ | ✓ | ✓ | X | ✓ | ✓ | ✓ | ✓ | ✓ | ✓ | ✓ | ✓ | ✓ | X | ✓ | X | ✓ |

| Criteria | Koskenvuo et al 2008 | Kunin-Baston et al 2015 | Lampe et al 2015 | Landolt et al 2012 | Laney et al 2010 | Langman et al 2014 | Maia et al 2015 | Martinuzzi et al 2007 | Martinuzzi et al 2008 | Masebas et al 2010 | Mata et al 2012 | McGovern et al 2008 | Mehta et al 2009 | Meiser et al 2005 | Millward et al 2001 | Miners et al 2002 | Modersitzki et al 2014 | Mundy et al 2006 | Munguaizquierdo et al 2015 | Neto et al 2018 | Oder et al 2016 | Okano et al 2013 |
| --- | --- | --- | --- | --- | --- | --- | --- | --- | --- | --- | --- | --- | --- | --- | --- | --- | --- | --- | --- | --- | --- | --- |
| Objectives clearly stated | ✓ | X | ✓ | ✓ | ✓ | ✓ | ✓ | X | ✓ | ✓ | ✓ | ✓ | X | ✓ | ✓ | ✓ | X | X | ✓ | ✓ | ✓ | ✓ |
| HrQoL as primary outcome | ✓ | ✓ | ✓ | ✓ | X | X | ✓ | NR | X | ✓ | ✓ | X | X | ✓ | ✓ | ✓ | ✓ | X | ✓ | ✓ | X | ✓ |
| Instrument selection justified | X | X | X | X | ✓ | X | ✓ | X | ✓ | X | ✓ | X | X | ✓ | ✓ | X | X | X | ✓ | X | ✓ | ✓ |
| Validated instrument | X | ✓ | ✓ | ✓ | ✓ | ✓ | ✓ | ✓ | ✓ | ✓ | ✓ | ✓ | ✓ | ✓ | ✓ | ✓ | ✓ | ✓ | ✓ | ✓ | ✓ | ✓ |
| Study design described | ✓ | ✓ | ✓ | ✓ | ✓ | ✓ | ✓ | X | ✓ | ✓ | ✓ | ✓ | ✓ | ✓ | X | ✓ | ✓ | X | ✓ | ✓ | ✓ | ✓ |
| Sampling method described | X | ✓ | ✓ | ✓ | ✓ | X | NA | X | ✓ | ✓ | ✓ | X | ✓ | ✓ | ✓ | ✓ | ✓ | NA | ✓ | ✓ | ✓ | X |
| Inclusion criteria described | X | X | ✓ | ✓ | ✓ | ✓ | ✓ | ✓ | ✓ | X | ✓ | ✓ | X | X | ✓ | X | ✓ | X | ✓ | ✓ | X | X |
| Excludes relevant individuals | NA | NA | ✓ | ✓ | X | X | ✓ | ✓ | ✓ | NA | ✓ | ✓ | NA | NA | ✓ | NA | ✓ | NA | ✓ | ✓ | NA | NA |
| Demographics described | ✓ | ✓ | ✓ | ✓ | ✓ | ✓ | ✓ | X | ✓ | ✓ | ✓ | ✓ | ✓ | ✓ | ✓ | X | ✓ | ✓ | X | ✓ | ✓ | ✓ |
| Clinical features described | ✓ | X | ✓ | X | X | X | ✓ | X | X | X | X | ✓ | ✓ | ✓ | X | ✓ | ✓ | X | X | X | ✓ | X |
| Sample size justified | X | X | X | NR | X | X | X | X | X | X | X | X | X | X | X | ✓ | ✓ | X | ✓ | ✓ | X | X |
| Self-completion by participants | NR | ✓ | ✓ | ✓ | ✓ | NR | NR | NR | ✓ | ✓ | ✓ | ✓ | NR | ✓ | ✓ | ✓ | ✓ | ✓ | ✓ | ✓ | NR | ✓ |
| Assessment time described | X | ✓ | ✓ | X | X | ✓ | X | X | ✓ | ✓ | X | X | ✓ | ✓ | X | ✓ | ✓ | ✓ | ✓ | ✓ | ✓ | X |
| Response rate reported | X | ✓ | X | ✓ | ✓ | X | ✓ | X | ✓ | ✓ | ✓ | X | ✓ | ✓ | ✓ | ✓ | X | ✓ | ✓ | ✓ | X | X |
| Loss to follow-up reported | ✓ | X | NA | NA | NA | X | NA | X | ✓ | NA | NA | NA | X | ✓ | ✓ | NA | NA | X | NA | NA | ✓ | NA |
| Missing data reported | X | X | X | X | X | X | X | X | X | ✓ | X | X | X | X | X | X | X | X | ✓ | X | X | X |
| Appropriate statistical analysis | ✓ | X | ✓ | ✓ | ✓ | ✓ | ✓ | X | ✓ | ✓ | ✓ | X | ✓ | ✓ | ✓ | ✓ | ✓ | X | ✓ | ✓ | ✓ | ✓ |
| Study findings described | ✓ | ✓ | ✓ | ✓ | ✓ | ✓ | ✓ | ✓ | ✓ | ✓ | ✓ | ✓ | ✓ | ✓ | ✓ | ✓ | ✓ | ✓ | ✓ | ✓ | X | ✓ |
| Study limitations described | ✓ | ✓ | ✓ | ✓ | ✓ | ✓ | X | ✓ | X | X | ✓ | ✓ | ✓ | ✓ | X | ✓ | ✓ | X | ✓ | ✓ | X | X |

| Criteria | Oliveira et al 2012 | Orlikowski et al 2011 | Packman et al 2007 | Pastores et al 2003 | Péntek et al 2016 | Pisani et al 2013 | Raluy-Callado et al 2013 | Regnery et al 2012 | Ries et al 2005 | Roe et al 2012 | Rombout-Sestrienkova et al 2015 | Schaefer et al 2015 | Sechi et al 2013 | Shapiro et al 2016 | Simon et al 2008 | Smid et al 2011 | Splinter et al 2015 | Storch et al 2008 | Street et al 2006 | Strohotte et al 2010 | Sutcliffe et al 2003 |
| --- | --- | --- | --- | --- | --- | --- | --- | --- | --- | --- | --- | --- | --- | --- | --- | --- | --- | --- | --- | --- | --- |
| Objectives clearly stated | ✓ | ✓ | ✓ | X | ✓ | ✓ | ✓ | X | ✓ | X | ✓ | ✓ | ✓ | ✓ | ✓ | ✓ | ✓ | ✓ | ✓ | ✓ | ✓ |
| HrQoL as primary outcome | ✓ | X | X | X | X | X | ✓ | X | ✓ | X | X | ✓ | ✓ | ✓ | ✓ | X | ✓ | ✓ | ✓ | X | NR |
| Instrument selection justified | X | X | X | X | ✓ | X | ✓ | X | X | X | X | X | ✓ | X | ✓ | X | X | ✓ | X | ✓ | X |
| Validated instrument | ✓ | ✓ | ✓ | ✓ | ✓ | ✓ | ✓ | ✓ | ✓ | ✓ | ✓ | ✓ | ✓ | ✓ | ✓ | ✓ | ✓ | ✓ | ✓ | ✓ | ✓ |
| Study design described | ✓ | X | ✓ | X | ✓ | ✓ | ✓ | ✓ | ✓ | ✓ | ✓ | ✓ | ✓ | ✓ | ✓ | ✓ | ✓ | X | ✓ | ✓ | X |
| Sampling method described | ✓ | ✓ | ✓ | ✓ | ✓ | ✓ | ✓ | ✓ | ✓ | X | ✓ | ✓ | ✓ | ✓ | ✓ | NA | ✓ | ✓ | ✓ | ✓ | NA |
| Inclusion criteria described | ✓ | ✓ | X | X | X | ✓ | ✓ | X | X | X | ✓ | ✓ | X | ✓ | ✓ | ✓ | ✓ | ✓ | X | ✓ | ✓ |
| Excludes relevant individuals | ✓ | X | NA | NA | NA | X | X | NA | NA | NA | X | ✓ | NA | ✓ | ✓ | ✓ | ✓ | ✓ | NA | X | X |
| Demographics described | X | ✓ | ✓ | ✓ | ✓ | ✓ | ✓ | ✓ | X | ✓ | ✓ | ✓ | ✓ | X | X | ✓ | X | ✓ | ✓ | ✓ | ✓ |
| Clinical features described | X | ✓ | X | ✓ | X | X | X | ✓ | ✓ | ✓ | ✓ | ✓ | ✓ | X | X | X | X | ✓ | X | ✓ | X |
| Sample size justified | X | X | X | X | X | X | X | X | X | X | ✓ | X | X | X | X | X | X | X | X | X | X |
| Self-completion by participants | NR | ✓ | ✓ | ✓ | ✓ | NR | X | ✓ | ✓ | ✓ | ✓ | ✓ | ✓ | ✓ | ✓ | NR | ✓ | ✓ | ✓ | ✓ | ✓ |
| Assessment time described | X | ✓ | X | ✓ | ✓ | ✓ | ✓ | ✓ | X | ✓ | ✓ | X | X | ✓ | ✓ | ✓ | ✓ | X | ✓ | ✓ | X |
| Response rate reported | X | ✓ | ✓ | X | X | X | X | ✓ | X | ✓ | ✓ | X | ✓ | X | ✓ | X | X | X | X | ✓ | ✓ |
| Loss to follow-up reported | NA | X | NA | NA | NA | X | NA | ✓ | NA | ✓ | ✓ | X | NA | NA | NA | X | NA | NA | NA | ✓ | ✓ |
| Missing data reported | X | X | X | X | X | X | X | X | X | X | X | X | ✓ | ✓ | X | X | X | X | X | X | X |
| Appropriate statistical analysis | ✓ | X | ✓ | X | X | X | X | ✓ | ✓ | X | ✓ | ✓ | ✓ | ✓ | ✓ | ✓ | X | ✓ | ✓ | X | ✓ |
| Study findings described | ✓ | ✓ | ✓ | ✓ | ✓ | X | ✓ | ✓ | ✓ | ✓ | ✓ | ✓ | ✓ | ✓ | ✓ | ✓ | ✓ | ✓ | ✓ | ✓ | ✓ |
| Study limitations described | ✓ | ✓ | X | X | ✓ | ✓ | ✓ | ✓ | ✓ | X | X | ✓ | X | ✓ | ✓ | ✓ | ✓ | ✓ | ✓ | X | ✓ |

| Criteria | Svetel el tal 2011 | Thimm et al 2013 | Tsuboi and Yamamoto 2012 | Tsuboi et al 2014 | Ulmer et al 2009 | van de Hilst el al 2008 | Van de Ploeg et al 2010 | Verhaak et al 2016 | Vielhaber et al 2011 | Vockley et al 2017 | Wagner et al 2014 | Wang et al 2007 | Watt et al 2010 | Weber et al 2016 | Weinreb et al 2007 | Wenzel et al 2007 | Wilcox et al 2008 | Wyatt et al 2012 | Zuraw et al 2011 |
| --- | --- | --- | --- | --- | --- | --- | --- | --- | --- | --- | --- | --- | --- | --- | --- | --- | --- | --- | --- |
| Objectives clearly stated | ✓ | ✓ | ✓ | ✓ | ✓ | ✓ | X | ✓ | X | ✓ | ✓ | ✓ | ✓ | ✓ | ✓ | ✓ | X | ✓ | ✓ |
| HrQoL as primary outcome | ✓ | ✓ | X | X | ✓ | X | X | ✓ | X | X | ✓ | NR | ✓ | X | X | X | NR | ✓ | ✓ |
| Instrument selection justified | ✓ | ✓ | X | X | X | ✝ | X | X | X | X | X | ✓ | ✓ | X | X | ✓ | X | ✓ | ✓ |
| Validated instrument | ✓ | ✓ | ✓ | ✓ | ✓ | ✓ | ✓ | ✓ | ✓ | ✓ | ✓ | ✓ | ✓ | ✓ | ✓ | ✓ | ✓ | ✓ | ✝ |
| Study design described | ✓ | ✓ | ✓ | ✓ | ✓ | ✓ | ✓ | ✓ | X | ✓ | ✓ | ✓ | ✓ | ✓ | ✓ | ✓ | ✓ | ✓ | ✓ |
| Sampling method described | NA | ✓ | NA | NA | NA | ✓ | ✓ | ✓ | X | ✓ | ✓ | X | ✓ | ✓ | ✓ | ✓ | NA | ✓ | ✓ |
| Inclusion criteria described | ✓ | ✓ | ✓ | ✓ | ✓ | ✓ | ✓ | ✓ | X | ✓ | ✓ | ✓ | ✓ | ✓ | ✓ | ✓ | ✓ | ✓ | X |
| Excludes relevant individuals | X | X | X | X | X | ✓ | X | X | NA | ✓ | X | X | X | X | ✓ | X | X | ✓ | NA |
| Demographics described | ✓ | ✓ | ✓ | ✓ | ✓ | ✓ | ✓ | ✓ | ✓ | X | ✓ | ✓ | ✓ | ✓ | ✓ | ✓ | ✓ | ✓ | ✓ |
| Clinical features described | ✓ | X | X | X | X | ✓ | ✓ | X | ✓ | ✓ | ✓ | ✓ | ✓ | ✓ | ✓ | X | X | X | X |
| Sample size justified | X | X | X | X | X | X | ✓ | X | X | ✓ | X | X | X | X | X | X | X | X | X |
| Self-completion by participants | ✓ | ✓ | NR | NR | ✓ | ✓ | ✓ | ✓ | ✓ | ✓ | ✓ | NR | ✓ | ✓ | NR | ✓ | NR | ✓ | ✓ |
| Assessment time described | X | ✓ | ✓ | ✓ | X | X | ✓ | ✓ | ✓ | ✓ | ✓ | X | ✓ | ✓ | ✓ | X | ✓ | X | X |
| Response rate reported | ✓ | ✓ | ✓ | X | ✓ | ✓ | X | ✓ | ✓ | X | ✓ | X | ✓ | X | ✓ | ✓ | ✓ | ✓ | ✓ |
| Loss to follow-up reported | ✓ | NA | ✓ | ✓ | ✓ | ✓ | ✓ | ✓ | ✓ | ✓ | ✓ | X | ✓ | NA | ✓ | ✓ | NA | ✓ | NA |
| Missing data reported | ✓ | ✓ | ✓ | ✓ | X | ✓ | X | X | X | X | ✓ | X | X | X | ✓ | ✓ | X | X | X |
| Appropriate statistical analysis | ✓ | ✓ | ✓ | ✓ | ✓ | ✓ | ✓ | ✓ | X | ✓ | ✓ | ✓ | ✓ | ✓ | ✓ | ✓ | ✓ | ✓ | ✓ |
| Study findings described | ✓ | ✓ | ✓ | ✓ | ✓ | ✓ | ✓ | ✓ | ✓ | ✓ | ✓ | ✓ | ✓ | ✓ | ✓ | ✓ | ✓ | ✓ | ✓ |
| Study limitations described | ✓ | X | ✓ | ✓ | ✓ | ✓ | ✓ | ✓ | ✓ | X | ✓ | ✓ | ✓ | ✓ | ✓ | X | ✓ | ✓ | ✓ |

Legend: ✓ - The study meets the criterium. X – The study does not meet the criterium; NA – Non-adequate: loss to follow-up does not apply in cross-sectional studies; we could not infer about exclusion of relevant individuals without reported inclusion criteria; NR – Not reported; ✝ - At least one validated instrument was used. Note: biochemical/laboratorial parameters were not considered as clinical features.
